# 3,14-Diselena-4,5,12,13-tetra­aza­tri­cyclo­[9.3.0.0^2,6^]tetra­deca-1(11),2(6),4,12-tetra­ene

**DOI:** 10.1107/S2414314626000635

**Published:** 2026-01-27

**Authors:** Heiner Detert, Dieter Schollmeyer

**Affiliations:** a University Mainz, Duesbergweg 10-14, 55099 Mainz, Germany; Goethe-Universität Frankfurt, Germany

**Keywords:** crystal structure, heterocycle, selenium, medium-sized ring

## Abstract

The crystal structure of the third isomer of cyclooctenobis-1,2,3-selenadiazoles, C_8_H_8_N_4_Se_2_, is reported. The mol­ecule is located on a twofold rotation axis. The eight-membered ring adopts a twist-chair conformation with planar heterocycles. C—H⋯N hy­dro­gen bonds connect the mol­ecules into layers parallel to (101).

## Abstract 2

A third isomer of cyclooctenobis-1,2,3-selenadiazoles is reported. The mol­ecule is located on a twofold rotation axis and the eight-membered ring adopts a twist-chair conformation with planar heterocycles.

## Graphical abstract

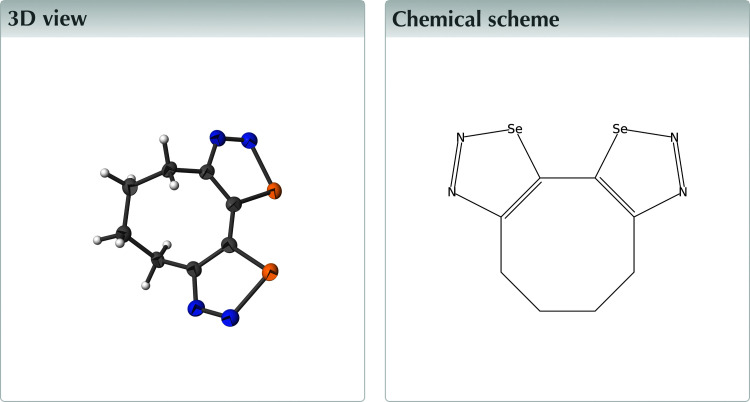

## Structure description

**Figure 1:**
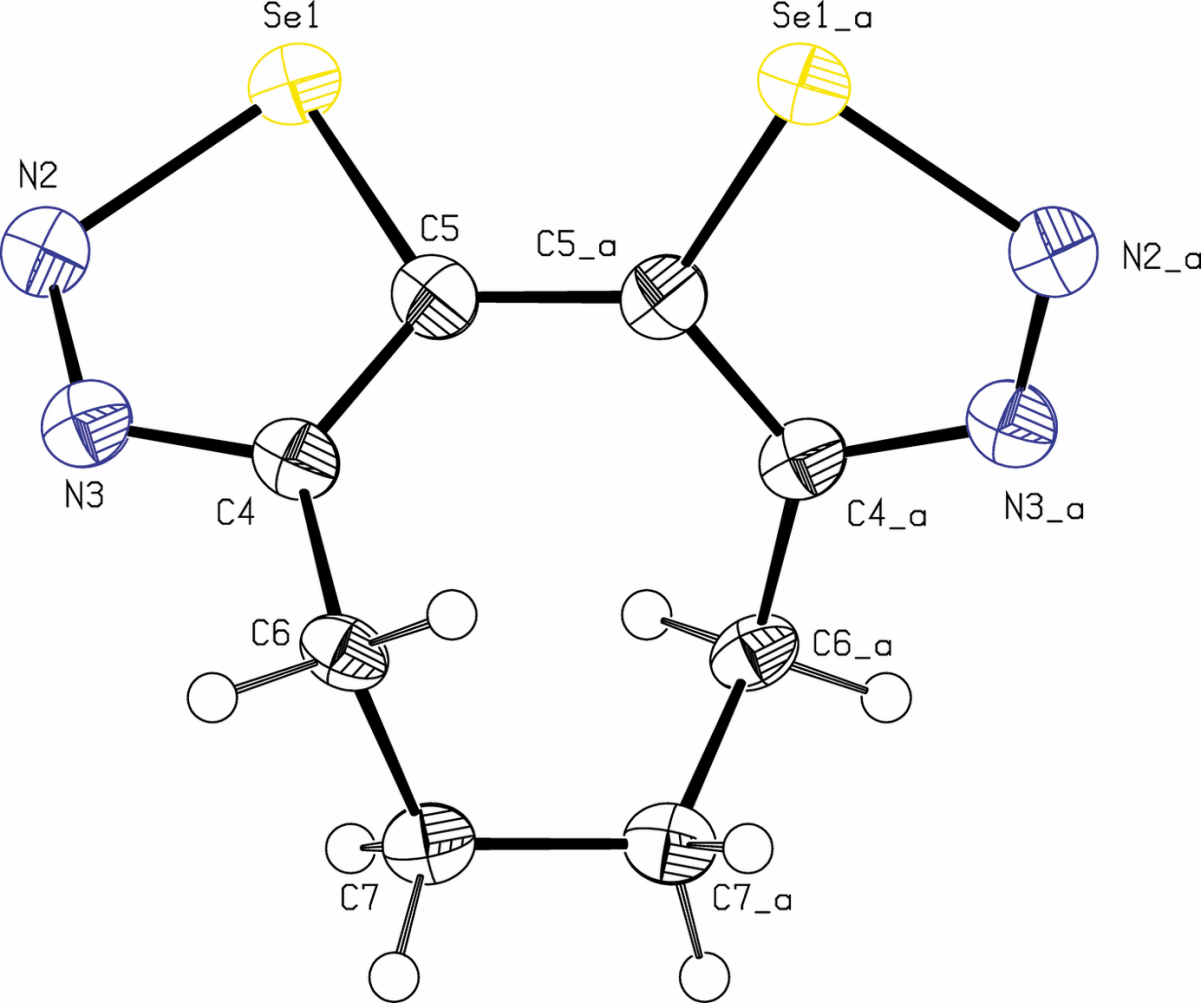
The mol­ecular structure of the title com­pound. Displacement ellipsoids are drawn at the 50% probability level.

**Figure 2:**
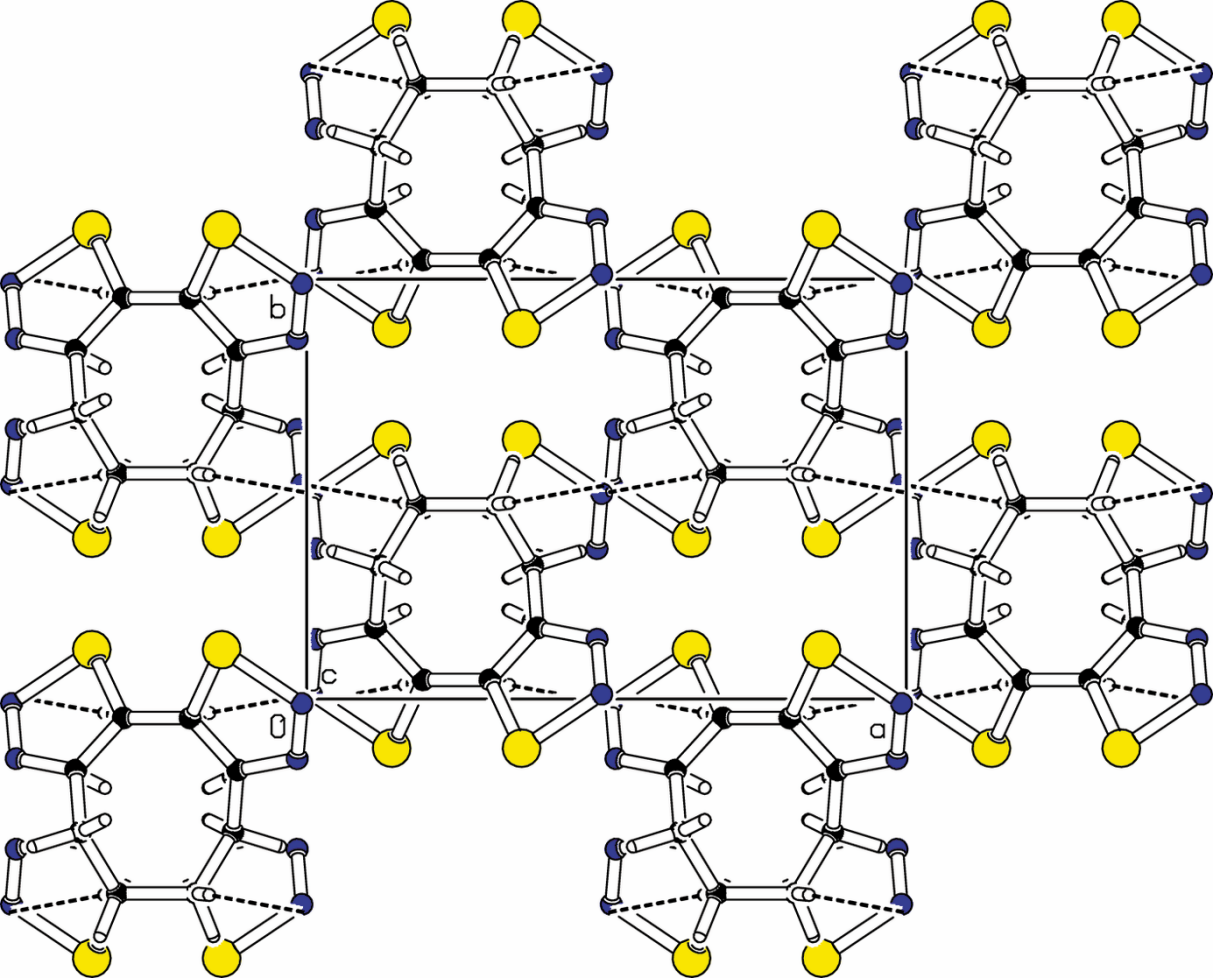
Part of the packing diagram, viewed along the *c*-axis direction.

The title com­pound, C_8_H_8_N_4_Se_2_ (Fig. 1 ▸), is the third isomer in the series of cyclo­octenobis-1,2,3-selena­diazo­les (Detert & Schollmeyer, 2020 ▸; Schollmeyer & Detert, 2025 ▸). These were prepared as part of a project on medium-sized cyclo­alkynes with functional and sterically demanding groups (Bissinger *et al.*, 1988 ▸; Detert *et al.*, 1994 ▸; Detert & Meier, 1997 ▸). Bis-1,2,3-selena­diazo­les are anti­microbial agents (Al-Smadi *et al.*, 2008 ▸) and important sources for medium-sized cyclo­alkadiynes (Gleiter *et al.*, 1988 ▸; Morales & Fronczek, 1994 ▸). The mol­ecule is *C*_2_-symmetric, having the eight-membered ring in a nearly twist-chair conformation with staggered C—H bonds. Both selena­diazole rings are planar within 0.02 (6) Å at atom C5 and the torsion angle between the heterocycles is 55.5 (3)°. This is significantly larger than the torsion angle [−43.5 (11)°] between the selena­diazole rings in the isomeric mol­ecule with an inverted orientation of the annulated heterocycles (Detert & Schollmeyer, 2020 ▸), probably due to the large atomic radii of the vicinal Se atoms in the title com­pound. The unit cell contains four mol­ecules connected *via* four hy­dro­gen bonds [C7—H7*b*⋯N2^i^; symmetry code: (i) −*x*, *y* + 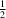, −*z* + 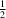]. The hy­dro­gen bonds (Table 1 ▸, Fig. 2 ▸) connect the mol­ecules to form layers parallel to (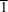
01).

## Synthesis and crystallization

The title com­pound appeared in the synthesis of its homo-conjugated isomer (Schollmeyer & Detert, 2025 ▸) from 1,4-cyclo­octa­nedione bis­semicarbazone and selenium dioxide in 1,4-dioxane in 12% yield. Recrystallization from a solution in chloro­form/ligroin gave brownish crystals (m.p. 393 K). IR (KBr): 2900, 2840, 1515, 1460, 1440, 1430, 1340, 1295, 1275, 1250, 1215, 945, 880, 850 cm^−1^. ^1^H NMR (400 MHz, CDCl_3_): δ 3.20 (*bs*, 4H), 1.90 (*bs*, 4H); ^13^C NMR (100 MHz, CDCl_3_): δ 158.8, 144.9 (C-1,2,7,10), 27.4, 25.1 (C-6,7,8,9); ^77^Se NMR (73 MHz, CDCl_3_, SeO_2_/D_2_O as reference): δ 295.9; ^15^N NMR (40,5 MHz, CDCl_3_, HC_3_NO_2_ as reference = 0): δ 93.9, 93.1; UV (EtOH, λ, logɛ): 207 (3.85), 220 (3.86), 261 (3.57), 302 (*sh*, 3.33), 339 nm (*sh*, 3.12); MS (FD): 290 (*M* – N_2_^+.^).

## Refinement

Crystal data, data collection and structure refinement details are summarized in Table 2 ▸. H atoms were placed at calculated positions and refined in the riding-model approximation, with C—H = 0.99 Å and *U*_iso_(H) = 1.2*U*_eq_(C).

## Supplementary Material

Crystal structure: contains datablock(s) I, global. DOI: 10.1107/S2414314626000635/bt4196sup1.cif

Structure factors: contains datablock(s) I. DOI: 10.1107/S2414314626000635/bt4196Isup2.hkl

Supporting information file. DOI: 10.1107/S2414314626000635/bt4196Isup3.cml

CCDC reference: 2524974

Additional supporting information:  crystallographic information; 3D view; checkCIF report

## Figures and Tables

**Table 1:** Hydrogen-bond geometry (Å, °)

*D*—H⋯*A*	*D*—H	H⋯*A*	*D*⋯*A*	*D*—H⋯*A*
C7—H7*B*⋯N2^i^	0.99	2.61	3.466 (9)	144

Symmetry code: (i) 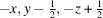.

**Table 2:** Experimental details

Crystal data	
Chemical formula	C_8_H_8_N_4_Se_2_
*M*_r_	318.10
Crystal system, space group	Monoclinic, *I*2/*a*
Temperature (K)	120
*a*, *b*, *c* (Å)	11.746 (2), 8.1617 (10), 10.0922 (15)
β (°)	97.121 (14)
*V* (Å^3^)	960.1 (3)
*Z*	4
Radiation type	Mo *K*α
μ (mm^−1^)	7.66
Crystal size (mm)	0.48 × 0.09 × 0.04

Data collection	
Diffractometer	STOE IPDS 2T
Absorption correction	Integration
*T*_min_, *T*_max_	0.468, 0.893
No. of measured, independent and observed [*I* > 2σ(*I*)] reflections	2481, 1144, 930
*R*_int_	0.037
(sin θ/λ)_max_ (Å^−1^)	0.663

Refinement	
*R*[*F*^2^ > 2σ(*F*^2^)], *wR*(*F*^2^), *S*	0.056, 0.148, 1.10
No. of reflections	1144
No. of parameters	64
H-atom treatment	H-atom parameters constrained
Δρ_max_, Δρ_min_ (e Å^−3^)	1.04, −1.75

Computer programs: *X-AREAWinXpose* (Stoe & Cie, 2020 ▸), *X-AREARecipe* (Stoe & Cie, 2020 ▸), *X-AREAIntegrate* (Stoe & Cie, 2020 ▸), *SHELXT2014* (Sheldrick, 2015*a* ▸), *SHELXL2019* (Sheldrick, 2015*b* ▸) and *PLATON* (Spek, 2020 ▸).

## full crystallographic data

### 3,14-Diselena-4,5,12,13-tetraazatricyclo[9.3.0.02,6]tetradeca-1(11),2(6),4,12-tetraene Crystal data

**Table:**

C_8_H_8_N_4_Se_2_	*F*(000) = 608
*M**_r_* = 318.10	*D*_x_ = 2.201 Mg m^−^^3^
Monoclinic, *I*2/*a*	Mo *K*α radiation, λ = 0.71073 Å
*a* = 11.746 (2) Å	Cell parameters from 5444 reflections
*b* = 8.1617 (10) Å	θ = 3.1–28.2°
*c* = 10.0922 (15) Å	µ = 7.66 mm^−^^1^
β = 97.121 (14)°	*T* = 120 K
*V* = 960.1 (3) Å^3^	Plate, colorless
*Z* = 4	0.48 × 0.09 × 0.04 mm

### 3,14-Diselena-4,5,12,13-tetraazatricyclo[9.3.0.02,6]tetradeca-1(11),2(6),4,12-tetraene Data collection

**Table:**

STOE IPDS 2T diffractometer	930 reflections with *I* > 2σ(*I*)
Detector resolution: 6.67 pixels mm^-1^	*R*_int_ = 0.037
rotation method, ω scans	θ_max_ = 28.1°, θ_min_ = 3.1°
Absorption correction: integration	*h* = −15→13
*T*_min_ = 0.468, *T*_max_ = 0.893	*k* = −10→10
2481 measured reflections	*l* = −13→13
1144 independent reflections	

### 3,14-Diselena-4,5,12,13-tetraazatricyclo[9.3.0.02,6]tetradeca-1(11),2(6),4,12-tetraene Refinement

**Table:**

Refinement on *F*^2^	Primary atom site location: dual
Least-squares matrix: full	Hydrogen site location: inferred from neighbouring sites
*R*[*F*^2^ > 2σ(*F*^2^)] = 0.056	H-atom parameters constrained
*wR*(*F*^2^) = 0.148	*w* = 1/[σ^2^(*F*_o_^2^) + (0.0748*P*)^2^ + 11.7676*P*] where *P* = (*F*_o_^2^ + 2*F*_c_^2^)/3
*S* = 1.10	(Δ/σ)_max_ < 0.001
1144 reflections	Δρ_max_ = 1.04 e Å^−^^3^
64 parameters	Δρ_min_ = −1.75 e Å^−^^3^
0 restraints	

### 3,14-Diselena-4,5,12,13-tetraazatricyclo[9.3.0.02,6]tetradeca-1(11),2(6),4,12-tetraene Special details

**Table:**

Geometry. All esds (except the esd in the dihedral angle between two l.s. planes) are estimated using the full covariance matrix. The cell esds are taken into account individually in the estimation of esds in distances, angles and torsion angles; correlations between esds in cell parameters are only used when they are defined by crystal symmetry. An approximate (isotropic) treatment of cell esds is used for estimating esds involving l.s. planes.

### 3,14-Diselena-4,5,12,13-tetraazatricyclo[9.3.0.02,6]tetradeca-1(11),2(6),4,12-tetraene Fractional atomic coordinates and isotropic or equivalent isotropic displacement parameters (Å^2^)

**Table:**

	*x*	*y*	*z*	*U*_iso_*/*U*_eq_	
Se1	0.14172 (6)	0.61729 (7)	0.33965 (6)	0.0274 (3)	
N2	0.0080 (5)	0.4890 (7)	0.3007 (5)	0.0291 (12)	
N3	0.0157 (5)	0.3578 (7)	0.3698 (5)	0.0289 (12)	
C4	0.1149 (6)	0.3307 (8)	0.4573 (6)	0.0259 (13)	
C5	0.1943 (5)	0.4546 (8)	0.4581 (6)	0.0269 (13)	
C6	0.1228 (6)	0.1830 (8)	0.5420 (6)	0.0254 (12)	
H6A	0.044169	0.148577	0.555241	0.031*	
H6B	0.163678	0.211823	0.630606	0.031*	
C7	0.1843 (6)	0.0368 (8)	0.4861 (6)	0.0284 (13)	
H7A	0.155565	−0.065021	0.523630	0.034*	
H7B	0.162450	0.033262	0.388198	0.034*	

### 3,14-Diselena-4,5,12,13-tetraazatricyclo[9.3.0.02,6]tetradeca-1(11),2(6),4,12-tetraene Atomic displacement parameters (Å^2^)

**Table:**

	*U*^11^	*U*^22^	*U*^33^	*U*^12^	*U*^13^	*U*^23^
Se1	0.0327 (4)	0.0269 (4)	0.0241 (4)	0.0027 (3)	0.0096 (3)	0.0013 (2)
N2	0.031 (3)	0.033 (3)	0.024 (2)	0.001 (2)	0.007 (2)	−0.001 (2)
N3	0.030 (3)	0.031 (3)	0.028 (3)	0.003 (2)	0.011 (2)	0.000 (2)
C4	0.027 (3)	0.027 (3)	0.026 (3)	−0.003 (3)	0.012 (2)	−0.003 (2)
C5	0.027 (3)	0.029 (3)	0.027 (3)	−0.001 (3)	0.011 (2)	−0.006 (2)
C6	0.029 (3)	0.024 (3)	0.025 (3)	−0.006 (3)	0.010 (2)	−0.002 (2)
C7	0.034 (3)	0.027 (3)	0.026 (3)	0.001 (3)	0.008 (3)	0.002 (3)

### 3,14-Diselena-4,5,12,13-tetraazatricyclo[9.3.0.02,6]tetradeca-1(11),2(6),4,12-tetraene Geometric parameters (Å, º)

**Table:**

Se1—C5	1.841 (7)	C6—C7	1.538 (9)
Se1—N2	1.888 (6)	C6—H6A	0.9900
N2—N3	1.275 (8)	C6—H6B	0.9900
N3—C4	1.390 (9)	C7—C7^i^	1.534 (13)
C4—C5	1.375 (9)	C7—H7A	0.9900
C4—C6	1.474 (9)	C7—H7B	0.9900
C5—C5^i^	1.467 (13)		
			
C5—Se1—N2	86.5 (3)	C7—C6—H6A	108.5
N3—N2—Se1	110.6 (5)	C4—C6—H6B	108.5
N2—N3—C4	118.6 (6)	C7—C6—H6B	108.5
C5—C4—N3	113.7 (6)	H6A—C6—H6B	107.5
C5—C4—C6	127.2 (6)	C7^i^—C7—C6	116.2 (5)
N3—C4—C6	119.1 (6)	C7^i^—C7—H7A	108.2
C4—C5—C5^i^	124.0 (5)	C6—C7—H7A	108.2
C4—C5—Se1	110.7 (5)	C7^i^—C7—H7B	108.2
C5^i^—C5—Se1	125.3 (3)	C6—C7—H7B	108.2
C4—C6—C7	114.9 (5)	H7A—C7—H7B	107.4
C4—C6—H6A	108.5		
			
C5—Se1—N2—N3	−0.1 (4)	C6—C4—C5—Se1	−177.1 (5)
Se1—N2—N3—C4	−0.1 (7)	N2—Se1—C5—C4	0.2 (4)
N2—N3—C4—C5	0.3 (8)	N2—Se1—C5—C5^i^	179.9 (6)
N2—N3—C4—C6	177.4 (5)	C5—C4—C6—C7	−87.5 (8)
N3—C4—C5—C5^i^	180.0 (6)	N3—C4—C6—C7	95.9 (7)
C6—C4—C5—C5^i^	3.2 (11)	C4—C6—C7—C7^i^	83.7 (8)
N3—C4—C5—Se1	−0.4 (7)		

Symmetry code: (i) −*x*+1/2, *y*, −*z*+1.

### 3,14-Diselena-4,5,12,13-tetraazatricyclo[9.3.0.02,6]tetradeca-1(11),2(6),4,12-tetraene Hydrogen-bond geometry (Å, º)

**Table:**

*D*—H···*A*	*D*—H	H···*A*	*D*···*A*	*D*—H···*A*
C7—H7*B*···N2^ii^	0.99	2.61	3.466 (9)	144

Symmetry code: (ii) −*x*, *y*−1/2, −*z*+1/2.
